# Reconstruction of bilateral inferomedial gluteal defects after resection of hidradenitis suppurativa with symmetrical keystone flaps designed parallel to relaxed skin-tension lines: A case report

**DOI:** 10.1097/MD.0000000000019779

**Published:** 2020-04-03

**Authors:** Hyo Bong Kim, Soo Yeon Lim, Chi Sun Yoon, Kyu Nam Kim

**Affiliations:** a Department of Plastic and Reconstructive Surgery, Konyang University Hospital, University of Konyang College of Medicine, Myunggok Medical Research Center, Daejeon; b Department of Plastic and Reconstructive Surgery, Wonkwang University Hospital, Wonkwang University School of Medicine, Iksan, Republic of Korea.

**Keywords:** gluteal defect, hidradenitis suppurativa, keystone flap, reconstruction, skin defect

## Abstract

**Rationale:**

Surgical treatment of hidradenitis suppurativa (HS) is challenging. Wide excision of affected lesions followed by skin graft or flap coverage has been recommended to achieve remission and prevent recurrence. Herein, we present our experience of bilateral inferomedial gluteal defects coverage using symmetrical keystone flaps (KFs) designed parallel to relaxed skin-tension lines (RSTLs).

**Patient concerns:**

An 18-year-old man was admitted for chronic inflammatory lesions in both inferomedial gluteal areas.

**Diagnoses:**

Physical examination revealed multiple sinuses with broad surrounding scars in both inferomedial gluteal areas, which led to a diagnosis of HS.

**Interventions:**

We performed wide excision on all affected lesions. The size of the final defects was 6 × 10 cm on the right side, and 5 × 9 cm on the left side. We covered the defects with KFs 9 × 15 cm (right) and 8 × 12 cm (left), which were based on the hotspots of the interior gluteal artery and internal pudendal artery perforators, and parallel to RSTLs.

**Outcomes:**

The flaps were inset without tension on each side, and the donor sites were closed primarily. All flaps fully survived and there were no postoperative complications. The patient was satisfied with the final outcome after 6 months of follow-up.

**Lessons:**

Successful reconstruction of bilateral inferomedial gluteal defects was achieved after resection of HS using symmetrical KFs designed parallel to RSTLs. The KF technique considering RSTLs could be a good reconstructive option for gluteal HS.

## 1 Introduction

Hidradenitis suppurativa (HS) is an inflammatory skin and subcutaneous tissue disease with a characteristic clinical presentation of chronically recurrent painful or suppurative lesions in apocrine gland-bearing areas.^[1,2]^ Global estimates of its prevalence vary between 0.03% and 4%, but the true prevalence of HS is unknown.^[3,4]^ The prevalence of HS in US and Australia is 0.10% and 0.67%, respectively, with women being more commonly affected in both countries.^[3,4]^ However, the prevalence of HS in Korea is 0.06%, with a predominance in men.^[5]^ The diagnosis of HS is made based on lesion morphology (nodules, abscesses, tunnels, and scars), location (intertriginous areas, such as axillae, inframammary folds, inguinal, gluteal folds, perineal, and perianal), and lesion progression (2 recurrences within 6 months or chronic or persistent lesions for more than 3 months).^[1]^ Surgical resection of all affected skin and soft tissue lesions is recommended as the only curative treatment to prevent recurrence and complications.^[1,2,6,7]^ This wide excision surgery inevitably leads to large defects, which should be covered with appropriate reconstructive modalities to achieve complete wound healing with favorable aesthetic and functional outcomes.^[2]^ There have been various reconstructive options for HS lesions, such as skin grafting, conventional fasciocutaneous (FC) flaps, and pedicled perforator flaps (PPFs).^[1,2,6–8]^ Among these, there is no universally accepted reconstructive method for gluteal defect coverage; therefore, reconstructive surgeons choose the proper method on a case-by-case basis. Herein, we present our experience of bilateral inferomedial gluteal defects reconstruction after resection of gluteal HS with symmetrical keystone flaps (KFs) designed parallel to relaxed skin-tension lines (RSTLs).

## 2 Case presentation

### 2.1 Case report

An 18-year-old man was admitted to our hospital for a 1-year history of chronic inflammatory skin lesions in both inferomedial gluteal areas. Although he had recurrent episodes of painful abscess and spontaneous pus-like discharge without any symptom of systemic inflammation, he took only anti-inflammatory drugs and dressed himself without visiting the hospital. He was a non-smoker and his body mass index was 18.17 kg/m^2^ (weight 55 kg, height 174 cm). He had no other pertinent medical history and no family history of HS. Physical examination revealed multiple sinuses with broad surrounding scars without active inflammation in both inferomedial gluteal areas. Laboratory tests of inflammatory markers including white blood cell count, C-reactive protein, and erythrocyte sedimentation rate were within normal range. We diagnosed his lesions as HS and planned surgical management.

### 2.2 Surgical procedures

**Figure 1:**
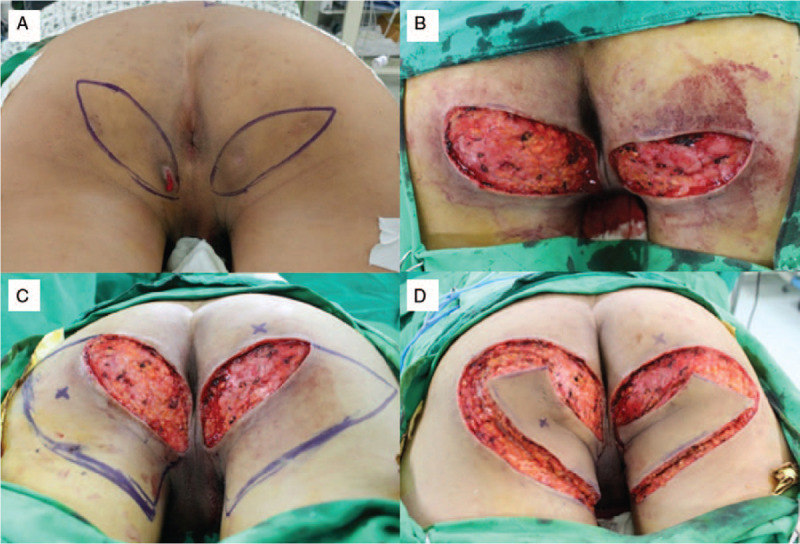
An 18-year-old man was admitted for chronic inflammatory lesions in both inferomedial gluteal areas. We diagnosed the condition as hidradenitis suppurativa. (A) Elliptical designs for wide excisions of affected lesions in both inferomedial gluteal areas. (B) The final defects after wide excisions were close to the anus (6 × 10 cm on the right side and 5 × 9 cm on the left side). (C) Designs of the keystone flaps (KFs) 9 × 15 cm (right) and 8 × 12 cm (left), based on the hotspots of the interior gluteal artery and internal pudendal artery perforators. The long axis of each flap was parallel to relaxed skin-tension lines (RSTLs). (D) Elevations of the Type IIA KFs with release of deep fascia and minimal undermining of flap margins.

**Figure 2:**
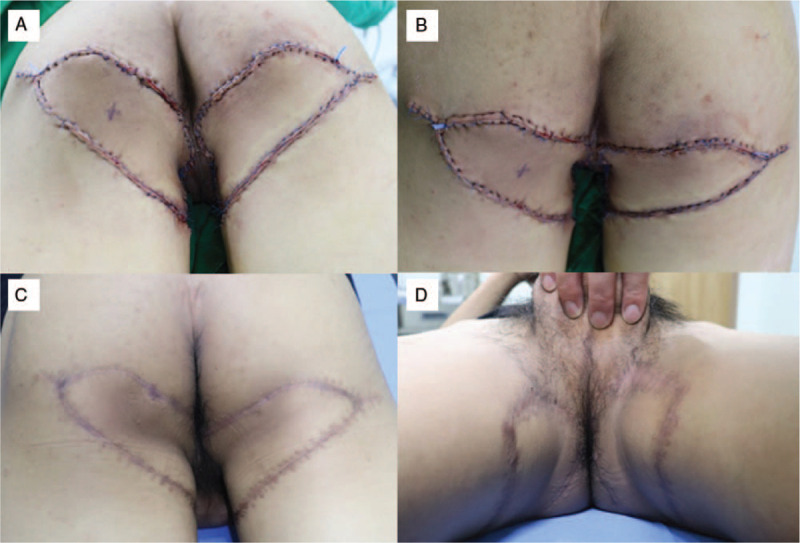
(A, B) Immediate postoperative photographs showed tension-free flap insetting and primary closure of the donor sites. (C, D) Postoperative photographs after 6 months of follow-up showed favorable outcomes, with the skin creases mimicking the natural creases and the scars hidden by the underpants.

We opted for a complete resection of all affected lesions and flap coverage (Fig. 1A). The operation was performed with the patient in the prone position under general anesthesia. We performed wide excision of the affected lesions and debridement of surrounding unhealthy tissues using the Versajet II hydrosurgery system (Smith and Nephew, St. Petersburg, 98 FL). The final post-debridement defects were 6 × 10 cm on the right side and 5 × 9 cm on the left side; the defects were very close to the anus medially (Fig. 1B). We symmetrically designed a 9 × 15 cm–sized KF (right side) and an 8 × 12 cm–sized KF (left side) based on the hot spots of the interior gluteal artery and internal pudendal artery perforators (Fig. 1C). At this time, we designed the long axis of each flap to be parallel to RSTLs in order to minimize wound tension and scar formation^[6]^ and the width of each flap was designed to be larger than the vertical width of the defect. Once the skin incision was made along the flap design, the dissection proceeded from the subcutaneous layer to the deep fascia. The fibrous subcutaneous septa and deep fascia were released using a monopolar device until the flaps could be moved freely from the surrounding tissues. After creating the island-shaped flap structure, the margin of the flaps was undermined minimally to preserve the integrity of the perforators (Fig. 1D). The procedure of insetting the flaps was first performed at its central portion on the side of the defect, and then on both ends, which were aligned in a V-Y apposition. Thus, both tension-free insetting of the flaps and primary closure of the donor sites were achieved (Fig. 2A and B). Mild compressive dressings were made with a foam material.

### 2.3 Outcomes

The total operative time was 185 minutes and the total flap elevation time was 39 minutes. The flaps completely survived without postoperative complications, such as hematoma, seroma, infection, or wound dehiscence. The patient was satisfied with the final outcome after 6 months of follow-up. The final scars were well hidden by the underpants and were tolerable without scar contracture, especially in the perianal area (Fig. 2C and D).

## 3 Discussion

We achieved a successful coverage of defects after wide resection of HS in both inferomedial gluteal areas using symmetrical KFs designed parallel to RSTLs, and good outcomes were obtained. Surgical treatment of HS is considered the best option for advanced and refractory HS, and can provide a shorter treatment period and lower recurrence than medical treatment.^[2,6,7]^ As aforementioned, wide resection of HS generally results in defect, and proper coverage is necessary. After surgical resection of gluteal HS, skin grafting has commonly been used to achieve a simple coverage of the defect.^[6,8]^ However, skin grafting for gluteal defects may often lead to contracture, stiffness, and contour deformity.^[2,6]^ Furthermore, skin grafting cannot provide enough cushions in these areas, and return to normal daily life is delayed due to a long healing time.^[2]^ However, flap coverage, in contrast to skin grafting, is associated with faster recovery, fewer complications, and more favorable outcomes.^[7]^ Free flap surgeries, including free transverse rectus abdominis myocutaneous flap, free latissimus dorsi myocutaneous flap, and free gluteal artery perforator flap, have been used for covering large and extensive gluteal defects.^[9–11]^ However, they may be regarded as overtreatment for small to moderate gluteal defects because there are generally enough surrounding tissues to apply as local flap in gluteal areas. Moreover, free flap surgery could be limited by the lack of skilled microsurgeons, the inability of centers to perform microsurgery, and inadequate postoperative microsurgical care.^[12]^ Moreover, difference in color, texture mismatch, and lack of sensation are potential problems in free flap reconstruction.^[12]^ Thus, local flaps are suitable for covering small to moderate defects in gluteal areas.

There have been various local flaps for covering gluteal defects after wide resection of HS, which include perforator-based FC flaps and various PPFs.^[2,6,7]^ A previous study presented 3 designs of FC flaps based on the inferior gluteal artery in accordance with the locations of defects after resection of gluteal HS as follows.^[6]^ Island V-Y advancement flap based on the descending branch of inferior gluteal artery was used for defects located on the upper part of the buttock; rotation V-Y advancement flap based on the first perforator of the deep femoral artery and the descending branch of the inferior gluteal artery was used for defects located lower part of the buttock; and bilobed flap including the descending and medial branches of the inferior gluteal artery in each lobe was used for perianal defects.^[6]^ These flaps are reliable for covering moderate to large gluteal defects, but have limitations in terms of flexibility in flap size and design, and rotation arc of the flap.^[2]^ Furthermore, postoperative scars are conspicuous because they are not parallel to skin creases and RSTLs. PPFs, such as superior and inferior gluteal artery perforator flaps, have been used for the reconstruction of gluteal, perineal, and perianal HS lesions of various sizes.^[2]^ Superior and inferior gluteal artery perforator flaps have long pedicles with a wide arc of rotation, which enable healthy tissue mobilization for a distance of up to 12 cm via intramuscular perforator dissection.^[2]^ These flaps as PPFs have superiority over conventional local flaps, including a flexible flap design with minimal constraints in the width-to-length ratio, lesser donor site morbidity, and providing a comparable like-with-like tissue for ideal reconstruction.^[12]^ However, microsurgical availability (intramuscular perforator dissection technique) is critical in PPF reconstruction, but may not be easily performed because all reconstructive surgeons cannot universally attain proficiency in microsurgical techniques.^[12]^

In our case, we performed the KF technique with consideration for RSTLs to cover inferomedial gluteal defects. The KF technique devised by Behan in 2003, a multi-perforator-based FC island flap with 2 conjoined V-Y flaps, has been commonly used to cover cutaneous defects at various anatomical locations because it has the advantages of a simple defect-adaptive design, easy reproducibility, reliable vascular perfusion, and safety.^[12–14]^ One of the inherent characteristics of the KF technique is minimal flap undermining and dissection; therefore, microsurgical pedicle dissection is not required unlike in the PPF technique.^[12,14]^ Namely, KF could be used as a good alternative to PPF and free flap in some circumstances like in our case due to its technical simplicity, ease, and safety. The principal biomechanics of KF reconstruction is the recruitment of tissue laxity.^[15,16]^ V-Y advancement flaps at either end of the KF facilitate the recruitment of laxity and skin tension is redistributed perpendicular to the direction of maximal wound tension.^[15,16]^ In our case, we designed each flap on the lower edge of the defect having greater tissue laxity, which can allow distribution of the tension required for closure throughout the periphery. Therefore, each flap was well maintained without any wound problem, such as wound dehiscence and breakage. Moreover, there was no scar contracture, especially in perianal areas. However, we designed the long axis of flap to be parallel to RSTLs, which aids to greatly reduce wound tension and scar formation. As a result, there was complete wound healing without complications in our case. Further, in contrast with perforator-based FC flap reconstruction,^[6]^ final scars were parallel to RSTL and well hidden by the underpants.

Although we achieved a successful KF reconstruction in gluteal HS, the present report has limitations. Our report included only 1 case. Therefore, further large-scale studies with prospective design will be required to confirm our outcome.

## 4 Conclusions

To the best of our knowledge, there has been no report of KF reconstruction for gluteal HS lesions. The current report presents the first successful case of bilateral inferomedial gluteal defects reconstruction after resection of gluteal HS with symmetrical KFs designed parallel to RSTLs. The KF technique considering the RSTL concept could be a good reconstructive option for gluteal HS in terms of low technical difficulty, safety, and favorable final scars.

## Acknowledgments

We would like to thank Editage (www.editage.com) for English language editing and publication support.

## Author contributions

**Conceptualization:** Chi Sun Yoon, Kyu Nam Kim.

**Data curation:** Hyo Bong Kim.

**Formal analysis:** Hyo Bong Kim, Soo Yeun Lim.

**Investigation:** Chi Sun Yoon, Kyu Nam Kim.

**Methodology:** Chi Sun Yoon, Kyu Nam Kim.

**Writing – original draft:** Hyo Bong Kim.

**Writing – review & editing:** Soo Yeun Lim, Chi Sun Yoon, Kyu Nam Kim.
